# Protein biomarkers associated with left bundle branch block in patients with heart failure and reduced ejection fraction

**DOI:** 10.1093/eschf/xvag009

**Published:** 2026-01-13

**Authors:** Karin Ljung, Christian Vestman, Ulrika Reistam, Frieder Braunschweig, Allan Zhao, Lars H Lund, Chim Lang, Adrian Voors, Eric Rullman, Gianluigi Savarese, Marcus Ståhlberg

**Affiliations:** Department of Medicine, Karolinska Institutet, Solna, Stockholm, Sweden; Department of Cardiology, Heart and Vascular Center, Karolinska University Hospital, Stockholm, Sweden; Department of Medicine, Karolinska Institutet, Solna, Stockholm, Sweden; Department of Cardiology, Heart and Vascular Center, Karolinska University Hospital, Stockholm, Sweden; Department of Medicine, Karolinska Institutet, Solna, Stockholm, Sweden; Department of Cardiology, Heart and Vascular Center, Karolinska University Hospital, Stockholm, Sweden; Department of Medicine, Karolinska Institutet, Solna, Stockholm, Sweden; Department of Cardiology, Heart and Vascular Center, Karolinska University Hospital, Stockholm, Sweden; Department of Physiology and Pharmacology, Karolinska Institutet, Stockholm, Sweden; Department of Medicine, Karolinska Institutet, Solna, Stockholm, Sweden; Department of Cardiology, Heart and Vascular Center, Karolinska University Hospital, Stockholm, Sweden; Division of Cardiovascular Research, School of Medicine, University of Dundee, Dundee, UK; Tuanku Muhriz Chair, Faculty of Medicine, National University of Malaysia, Bangi, Selangor, Malaysia; Department of Cardiology, University of Groningen, University Medical Center of Groningen, Groningen, The Netherlands; Department of Laboratory Medicine, Division of Clinical Physiology, Karolinska Institutet, Stockholm, Sweden; Unit of Clinical Physiology, Karolinska University Hospital, Stockholm, Sweden; Department of Clinical Science and Education, Södersjukhuset, Karolinska Institutet, Stockholm, Sweden; Department of Medicine, Karolinska Institutet, Solna, Stockholm, Sweden; Department of Cardiology, Heart and Vascular Center, Karolinska University Hospital, Stockholm, Sweden

**Keywords:** Heart failure, Remodelling, Proteomics, Biomarkers, Dyssynchrony, Left bundle branch block

## Abstract

**Introduction:**

Left bundle branch block (LBBB) is common in heart failure with reduced ejection fraction (HFrEF) and causes dyssynchrony, which accelerates cardiac remodelling. The biological mechanisms behind LBBB-associated remodelling remain largely unknown. Therefore, we used an omics approach to test the hypothesis that LBBB is associated with plasma protein dysregulation aiming at defining a proteome that contributes to the specific disease driving phenotype in dyssynchronopathy.

**Methods:**

Patients were selected from the BIOlogy Study to TAilored Treatment in Chronic Heart Failure database (*n* = 4254). Patients with HFrEF and LBBB (HFrEF + LBBB) served as cases (*n* = 268) and a matched control group (*n* = 268) with HFrEF without LBBB (HFrEF − LBBB) was selected using propensity score matching. We compared relative plasma concentrations of 364 proteins between the two groups using proximity extension assay.

**Results:**

HFrEF + LBBB was associated with up- or downregulation of 41 proteins out of 364 assessed, 11%, compared with HFrEF − LBBB. Fibroblast growth factor 2 (FGF2) decreased, epidermal growth factor receptor (EGFR) increased, and several cytokines and proteins involved in extracellular matrix processing changed expression. Gene ontology pathways enriched among upregulated proteins were mainly involved in immune response and cell signalling and included the mitogen-activated protein kinase (MAPK) pathway.

**Conclusion:**

In HFrEF, LBBB was associated with an altered circulating proteome. FGF2 and EGFR were highly dysregulated between the groups and the MAPK signalling pathway was affected, all of which may be pathophysiologically involved in the accelerated cardiac remodelling observed in these patients. These findings constitute a foundation for future studies of relevant LBBB-related biomarkers, treatment targets, and mechanisms behind the cardiac remodelling observed when LBBB is superimposed on HFrEF.

## Graphical Abstract

**Graphical Abstract:**
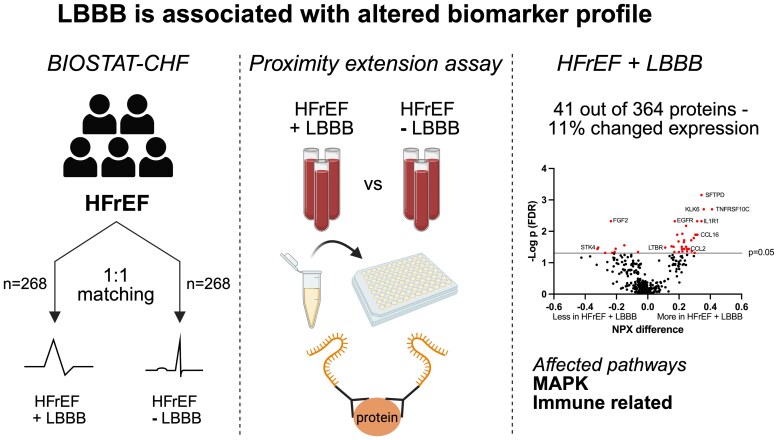
The graphical abstract was created in https://BioRender.com.

## Introduction

Heart failure with reduced ejection fraction (HFrEF) is a common cause of death in western countries^1^ and is characterized by left ventricular remodelling, i.e. left ventricular hypertrophy and dilatation.^2^ Pharmacological treatment, cardiac resynchronization therapy (CRT) and mechanical unloading may reverse maladaptive cardiac remodelling in HFrEF and the extent of reverse remodelling has been linked to improved clinical outcomes.^2^ Although remodelling and its reversal is central in HFrEF, the biological mechanisms governing this process remain incompletely understood. Approximately 30% of patients with HFrEF have concomitant left bundle branch block (LBBB), resulting in a dyssynchronous electrical and mechanical activation pattern.^3^ Left bundle branch block is associated with accelerated remodelling and a worsened prognosis in HFrEF.^3,4^ Experimental work in canine and mouse models has documented some unique features of dyssynchrony-induced remodelling: accelerated chamber dilatation,^5^ asymmetrical hypertrophy,^6^ changes in conduction velocities and gap junction properties,^7^ regional and global changes in gene and protein expression,^8–10^ reduced β-adrenergic signalling,^11^ reduced inotropy through Ca^2+^ desensitization,^12^ and decreased mitochondrial function.^13^ Although the remodelling mechanisms associated with dyssynchrony may be accompanied with change in the composition of blood plasma proteins in HFrEF with LBBB compared with HFrEF without LBBB, previous data on the biological imprint of dyssynchrony in patients with HFrEF are scarce and limited to the analysis of a small number of circulating proteins.^14,15^

Therefore, we used an omics approach to test the hypothesis that LBBB is associated with plasma protein dysregulation aiming at defining a proteome that contributes to the specific disease driving phenotype in dyssynchronopathy.

## Methods

### Patient population

We used data from the BIOlogy Study to TAilored Treatment in Chronic Heart Failure (BIOSTAT-CHF).^16^ The overall aim of the BIOSTAT-CHF study was to identify biological pathways associated with a response to titration of heart failure drugs according to current guidelines. Patient selection to BIOSTAT-CHF have been described in detail previously.^16^ In short, 4254 patients with new onset or worsening heart failure who had not yet received optimal medical treatment were recruited from European centres between December 2010 and April 2014. Heart failure diagnosis was determined either through ejection fraction (EF) ≤40% or an increase of brain natriuretic peptide (BNP), N-terminal pro-BNP (NT-proBNP), or previous hospitalization due to heart failure. Hence, the overall BIOSTAT-CHF population consists of a heart failure population across the spectrum of EF. Several clinical characteristics central to heart failure are registered in the BIOSTAT-CHF database.

Since the association between LBBB, accelerated remodelling, and a worse prognosis has only been established for HFrEF, we selected patients from BIOSTAT-CHF with LBBB and reduced EF (<40%) as cases (HFrEF + LBBB). Their protein biomarker profile was compared with propensity score–matched patients with HFrEF but without LBBB (HFrEF − LBBB) to find associations between LBBB and protein biomarkers in HFrEF.

### Patient selection

**Figure 1:**
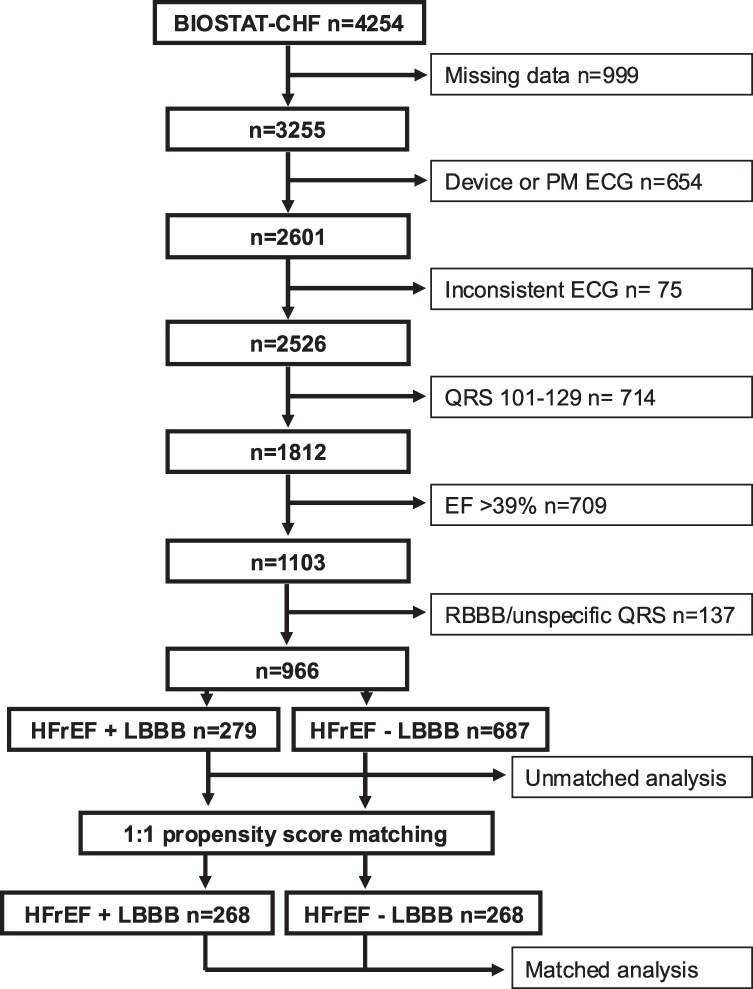
A flow chart describing the process of patient selection and on which grounds patients were excluded. Nine hundred and ninety-nine patients were excluded due to missing data, where ejection fraction, QRS duration, and measurements of biomarkers were missing. Six hundred and fifty-four patients were excluded due to pacemaker rhythm on electrocardiogram or an implanted device. Seventy-five patients had inconsistent electrocardiogram, and 714 had QRS between 101 and 129 ms, which lead to exclusion. Seven hundred and nine patients did not fulfill heart failure with reduced ejection fraction criteria due to ejection fraction >39% and were therefore excluded. One hundred and thirty-seven patients were excluded due to right bundle branch block or unspecific QRS appearance. After this selection, 966 patients were used for 1:1 propensity score matching, yielding 268 matched patients in each group. BIOSTAT-CHF, Biology Study to Tailored Treatment in Chronic Heart Failure; PM, pace-maker; ECG, electrocardiogram; EF, ejection fraction; RBBB, right bundle branch block; DHF, dyssynchronous heart failure; SHF, synchronous heart failure; HFrEF, heart failure with reduced ejection fraction

Patients were selected from the BIOSTAT-CHF database according to the flowchart presented in *Figure 1*. Patients were excluded if key variables were missing, electrocardiogram (ECG) showed paced QRS complexes, inconsistent ECG (e.g. LBBB but QRS duration <120 ms), QRS-duration 101–129 ms, right bundle branch block or unspecific QRS broadening. Also, patients with EF ≥40% were excluded.

The study population (*n* = 966) was divided into two groups: HFrEF + LBBB (*n* = 279, defined as EF < 40% and QRS > 130 ms with LBBB morphology) and HFrEF − LBBB (*n* = 687, defined as EF < 40% and QRS ≤100 ms).

### Blood sampling and protein analysis

Blood samples were collected at inclusion in BIOSTAT-CHF and after 9 months. For this study, only protein data from blood samples taken at inclusion were used. Blood samples were subsequently analysed for relative protein concentrations by means of proximity extension assay technology using Olink proseek Multiplex CVDII, CVDIII, ONC, and INF panels (Olink®, Thermo Fischer Scientific). This allowed for simultaneous measurement of 364 unique proteins in each sample. The panels were not normalized but log transformed. The CVDIII panel was measured in 2017 and not matched with the other. CVDII, ONC, and INF were measured together in 2019. We did not have missing values.

### Recalculated variables

Estimated glomerulus filtration rate (eGFR) was calculated from creatinine, age, and gender by using the modification of diet in renal disease (MDRD) formula. Mean arterial pressure (MAP) was calculated from systolic and diastolic blood pressure. Body mass index (BMI) was calculated from the weight and height. Patients were considered to have atrial fibrillation/flutter if they had atrial fibrillation/flutter in their medical history and/or on the ECG at admission. Heart failure aetiology was classified as ischaemic cardiomyopathy in patients with previous percutaneous coronary intervention (PCI), coronary artery bypass grafting, or myocardial infarction.

### Statistical analysis

#### Clinical data

Categorical variables were presented with *n* and percentage and tested comparing HFrEF + LBBB and HFrEF − LBBB using *χ*^2^ test. Continuous variables were described using median and interquartile range (IQR) and tested between HFrEF + LBBB and HFrEF − LBBB with Mann–Whitney *U* test.

#### Propensity score matching

To reduce the risk of bias due to differences between the groups in important heart failure and biomarker-related baseline characteristics, HFrEF − LBBB patients were propensity score matched (1:1) to HFrEF + LBBB patients based on 10 selected variables: eGFR, age, EF, BMI, sex, ischaemic aetiology, diabetes mellitus, atrial fibrillation/flutter, New York Heart Association (NYHA) class, and heart failure hospitalization during the previous year. The matching was performed with a tolerance level set to 0.03. When evaluating the effect of propensity score matching on smoothing out differences in baseline characteristic variables, a standard mean difference (SMD) < 0.2 was set as limit where variables were considered equal between the groups.

#### Protein data

Protein levels were expressed as normalized protein expression (NPX). Significance testing for differences in NPX for each protein was performed using Mann–Whitney *U* test. A false detection rate (FDR) of 5% was applied to adjust for multiple comparisons. A *q*-value (FDR adjusted *P*) < .05 was considered significant. All statistical analyses were performed using IBM SPSS statistics, version 27.

### Bioinformatics

The STRING database^17^ was used to analyse protein–protein interactions for proteins with significant differences in expression levels between HFrEF + LBBB and HFrEF − LBBB. To identify enriched biological pathways, we used the gene ontology (GO) biological processes and Kyoto Encyclopedia of Genes and Genomes (KEGG) from the STRING database. Up- and downregulated proteins were analysed separately to improve detection of relevant interactions.^18^

## Results

### Baseline characteristics and propensity score matching

Baseline characteristics before and after propensity score matching are shown in *Table 1*.

**Table 1:** Patient characteristics before and after propensity score matching

Variable	Unmatched		SMD	*P*-value	Matched		SMD	*P*-value
	HFrEF + LBBB (*n* = 279)	HFrEF − LBBB (*n* = 687)			HFrEF + LBBB (*n* = 268)	HFrEF − LBBB (*n* = 268)		
**Demographics**								
**Sex, male, % (*n*)**	67.7 (189)	71 (488)	0.086	.311 ^P^	67.5 (181)	66 (177)	0.037	.714^P^
**Age, years, median (IQR)**	74.3 (64.6–79.4)	66.7 (58.2–75.6)	0.475	<.001^M^	74.1 (64.4–79.3)	72.7 (64.9–78.9)	0.028	.564^M^
**Race, Caucasian, % (*n*)**	99.6 (278)	98.8 (679)	0.654	.126^P^	99.6 (267)	99.6 (267)	0	.365^P^
**BMI, kg/m^2^ median (IQR)**	26.6 (23.5–30.4)	27.2 (23.9–31.2)	0.128	.074^M^	26.6 (23.4–30.4)	26.4 (23.9–30.1)	0.014	.893^M^
**Weight, kg median (IQR)**	76 (67–88.3)	80 (68–93)	0.181	.007^M^	76 (67–88)	76.5 (66.3–90)	0.030	.565^M^
**Height, cm median (IQR)**	170 (163–176)	170 (164–178)	0.142	.086^M^	170 (163–176)	170 (163–177)	0.053	.660^M^
**NYHA Class, % (*n*)**			0.019	.949^P^			0.087	.749^P^
**I**	1.8 (5)	1.9 (13)			1.5 (4)	2.2 (6)		
**II**	43 (120)	41.9 (288)			43.7 (117)	41 (110)		
**III**	41.6 (116)	43.2 (297)			42.5 (114)	45.9 (123)		
**IV**	12.2 (34)	11.2 (77)			12.3 (33)	10.8 (29)		
**Cardiac ultrasound, median (IQR)**								
**LVEF**, **%**	28 (23–35)	30 (25–35)	0.184	.003^M^	28.5 (23–35)	27 (20–34)	0.087	.420^M^
**LVEDD, mm**	62 (57–69)	59 (54–65)	0.350	<.001^M^	62 (57–69)	60 (54–65)	0.287	.001^M^
**LVESD, mm**	54 (46–60)	49 (45–56)	0.338	.001^M^	53 (46–61)	49 (44–55)	0.352	.001^M^
**Clinical profile, median (IQR)**								
**Heart rate**, b.p.m.	74 (65–89)	80 (69–93)	0.255	<.001^M^	73 (65–89)	80 (68–95)	0.343	<.001^M^
**Mean artery pressure**, **mmHg**	88 (79–97)	92 (82–102)	0.299	<.001^M^	88 (79–96)	93 (83–104)	0.466	<.001^M^
**ECG rhythm, % (*n*)**								
**Sinus**	74.9 (209)	62.6 (430)	0.319	<.001^P^	73.9 (198)	64.2 (172)	0.252	.015^P^
**Atrial fibrillation/flutter**	22.9 (64)	35.5 (244)	0.339	<.001^P^	23.9 (64)	33.6 (90)	0.263	.013^P^
**Other**	2.2 (6)	1.9 (13)	0.072	.793^P^	2.2 (6)	2.2 (6)	0	1.0^P^
**QRS morphology**								
**LBBB, % (*n*)**	100 (279)	0 (0)	0.072	<.001^P^	100 (268)	0 (0)		<.001^P^
**QRS-duration, ms median (IQR)**	152 (140–164)	90 (80–96)	4.446	<.001^M^	152 (140–164)	90 (80–98)	4.459	<.001^M^
**Aetiology, % (*n*)**								
**Isch**a**emic cardiomyopathy**	47.3 (132)	41.9 (288)	0.120	.126^P^	47.4 (127)	42.5 (114)	0.108	.259^P^
**Non-isch**a**emic cardiomyopathy**	52.7 (147)	58.1 (399)	0.120	.126^P^	52.6 (141)	57.5 (154)	0.108	.259^P^
**Medical history, % (*n*)**								
**Hypertension**	44.1 (123)	57.6 (396)	0.041	.606^P^	55.6 (149)	61.9 (166)	0.145	.025^P^
**Atrial fibrillation/flutter**	34.4 (96)	44.3 (304)	0.228	.005^P^	35.8 (96)	39.6 (106)	0.088	.373^P^
**Myocardial infarction**	37.6 (105)	38.1 (262)	0.013	.871^P^	37.7 (101)	38.4 (103)	0.018	.859^P^
**PCI**	19 (53)	17.6 (121)	0.047	.640^P^	19 (51)	17.9 (48)	0.041	.738^P^
**CABG**	16.8 (47)	10.2 (70)	0.320	.004^P^	17.5 (47)	11.6 (31)	0.268	.050^P^
**Diabetes mellitus**	27.2 (76)	27.9 (192)	0.020	.824^P^	27.6 (74)	27.6 (74)	0	1.0^P^
**Peripheral artery disease**	13.3 (37)	9.9 (68)	0.186	.121^P^	13.1 (35)	10.1 (27)	0.166	.266^P^
**Stroke**	11.1 (31)	9.2 (63)	0.119	.353^P^	10.4 (28)	12.7 (34)	0.116	.436^P^
**COPD**	14.3 (40)	13 (89)	0.066	.559^P^	14.6 (39)	15.3 (41)	−0.03	.822^P^
**Previous HF hospitalization (prior year)**	28 (78)	24.5 (168)	0.095	.281^P^	28.7 (77)	30.6 (82)	0.049	.636^P^
**Lab median (IQR)**								
**NT-proBNP, ng/l**	2974 (1089–6370)	2686 (920–5476)	0.051	.229^M^	3006 (1127–6406)	4241 (2102–8527)	0.216	.008^M^
**H**a**emoglobin, g/dl**	13.3 (12–14.5)	13.7 (12.3–14.9)	0.156	.029^M^	13.4 (12–14.6)	13.4 (12–14.5)	0.015	.947^M^
eGFR, **ml/min/1.73m^2^**	60 (45–65)	60.0 (53–76)	0.352	<.001^M^	60 (46–65)	60 (47–77)	0.176	.049^M^
**Creatinine (**µ**mol/**l)*	105 (85–130)	94 (79–115)	0.222	<.001^M^	102.8 (85.0–127.6)	103.9 (84.3–128)	0.014	.891^M^
**Medical treatment, % (*n*)**								
**ACEi/ARB**	76 (212)	77.3 (531)	0.040	.662^P^	76.5 (205)	73.9 (198)	0.077	.484^P^
**Loop-diuretics**	99.6 (278)	99.7 (685)	0.115	.865^P^	99.6 (267)	99.6 (267)	0	1.0 ^P^
**Beta-blockers**	83.2 (232)	85.2 (585)	0.083	.436^P^	82.8 (222)	83.6 (224)	0.030	.817^P^
**MRA**	45.9 (128)	50.8 (349)	0.109	.165^P^	46.6 (125)	51.9 (139)	0.115	.226^P^

Patient characteristics comparing HFrEF + LBBB and HFrEF − LBBB before (unmatched) and after (matched) propensity score matching. Variables included in the model is marked in the table with *.

HFrEF, heart failure with reduced ejection fraction; LBBB, left bundle branch block; SMD, standardized mean difference; IQR, interquartile range; BMI, body mass index; NYHA, New York heart association; LVEF, left ventricular ejection fraction; LVEDD, left ventricular end diastolic diameter; LVESD, left ventricular end systolic diameter; b.p.m., beats per minute; ECG, electrocardiogram; PCI, percutaneous coronary intervention; CABG, coronary artery bypass grafting; COPD, chronic obstructive pulmonary disease; HF, heart failure; ACEi, angiotensin converting enzyme inhibitor; ARB, angiotensin receptor blocker; NT-proBNP, N-terminal pro-brain natriuretic peptide; eGFR, estimated glomerular filtration rate; MRA, mineralocorticoid receptor antagonist. ^P^Pearson-*χ*^2^. ^M^Mann–Whitney *U* test.

Prior to matching, some significant differences were found between the two groups: Patients with HFrEF + LBBB were older (74 vs 67 years), had lower left ventricular EF (LVEF) (28% vs 30%), lower prevalence of atrial fibrillation/flutter (22.9% vs 35.5%), and higher NT-proBNP (2974 vs 2686 ng/l) compared with the HFrEF − LBBB patients.

Propensity score matching reduced the differences in age (SMD = 0.03) and LVEF (SMD = 0.09) but the lower prevalence of atrial fibrillation remained in HFrEF + LBBB patients (23.9% vs 33.6%, SMD = 0.26). After propensity score matching, NT-proBNP was lower in HFrEF + LBBB patients (3006 vs 4241 ng/l, SMD = 0.22). Additionally, mean arterial pressure remained significantly lower in HFrEF + LBBB even after matching (88 vs 93 mmHg, SMD = 0.47). Evidence-based pharmacological treatment, which may influence biomarker profile did not differ significantly between groups before or after matching. All patients were treated with furosemide, almost 80% with β-blockers and ∼80% with angiotensin receptor blockers or angiotensin-converting enzyme inhibitors. Mineral corticoid receptor inhibitors were less common and used by 46.6% in the HFrEF + LBBB group and 51.9% in the HFrEF − LBBB group (*P* = .226) after matching, which was similar to before matching (45.9% vs 50.8% *P* = .165).

### Protein dysregulation in heart failure with left bundle branch block

**Figure 2:**
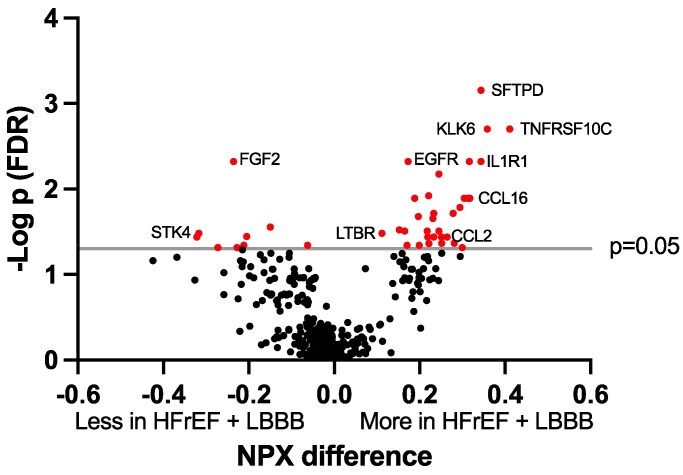
The volcano plot shows the differences in protein concentrations between HFrEF + LBBB and HFrEF − LBBB. The concentrations of 41 proteins, out of 364 analysed, differed significantly between the two groups and are indicated in red. The level of statistical significance, −log_10_*P* is given on the *y*-axis, and *P* = 0.05 (false detection rate 5%) is indicated with a grey vertical line. The normalized protein expression difference is given on the *x*-axis and was calculated as the normalized protein expression for a certain protein in blood samples from patients with HFrEF − LBBB deducted from the normalized protein expression for the same protein from patients with HFrEF + LBBB. Since the normalized protein expression is given in a log_2_ scale, a difference of 1 responds to a doubling in protein concentration. HFrEF, heart failure with Reduced ejection fraction; LBBB, left bundle branch block; FDR, false detection rate; NPX, normalized protein expression; SFTPD, surfactant protein D; KLK6, kallikrein 6; TNFRSF10C, decoy receptor 1; FGF2, fibroblast growth factor 2; EGFR, epidermal growth factor receptor; CCL2, C–C motif ligand 2; LTBR, lymphotoxin beta receptor; IL1R1, interleukin 1 receptor type 1; CCL16, chemokine C–C motif ligand 16; STK4, serine/threonine-protein kinase 4

From the BIOSTAT-CHF data, we retrieved the levels of 364 unique proteins. Among matched patients, 41 proteins (11%) had significantly different expression levels in HFrEF + LBBB compared with HFrEF − LBBB (32 upregulated and 9 downregulated) (*Figure 2*). *Table 2* lists all significantly dysregulated proteins in HFrEF + LBBB. Notably, several cytokines and chemokines were among the upregulated proteins in HFrEF + LBBB as well as proteins involved in extracellular matrix processes. Tables of all 364 proteins with *P*-values for matched or unmatched comparisons between HFrEF ± LBBB are available in Supplementary Tables S1 and S2.

**Table 2:** Proteins significantly changed in HFrEF + LBBB compared with HFrEF − LBBB

Higher concentration in HFrEF + LBBB	NPX difference	*P*-value
Surfactant protein D (PSP-D)	0.343	.001
Kallikrein 6 (KLK6)	0.359	.002
Decoy receptor 1 (TNFRSF10C)	0.411	.002
Interleukin 1 receptor type 1 (IL-1RT1)	0.317	.005
Epidermal growth factor receptor (EGFR)	0.173	.005
Matrix extracellular phosphoglycoprotein (MEPE)	0.344	.005
Vascular endothelial cadherin (CDH5)	0.245	.007
Intercellular adhesion molecule 2 (ICAM-2)	0.221	.012
Paraoxonase 3 (PON3)	0.318	.013
Chemokine C-X-C motif ligand 16 (CXCL16)	0.304	.013
Chemokine C-C motif ligand 16 (CCL16)	0.313	.013
Interleukin 1 receptor type 2 (IL-1RT2)	0.188	.013
Tartrate-resistant acid phosphatase type 5 (TR-AP)	0.295	.016
Protein delta homologue 1 (DLK-1)	0.279	.019
Tyrosine-protein kinase receptor UFO (AXL)	0.233	.019
Ephrin type B receptor 4 (EPHB4)	0.197	.021
CD166 antigen (ALCAM)	0.231	.022
Proprotein convertase subtilisin/kexin type 9 (PCSK9)	0.152	.030
Granulin (GRN)	0.165	.031
Tumour necrosis factor ligand superfamily member 13B (TNFSF13B)	0.218	.031
Trefoil factor 3 (TFF3)	0.244	.031
Lymphotoxin beta receptor (LTBR)	0.112	.033
Peptidoglycan recognition protein 1 (PGLYRP1)	0.233	.037
Spondin 1 (SPON1)	0.219	.037
Renin (REN)	0.264	.037
Cathepsin Z (CTSZ)	0.251	.037
Urokinase type plasminogen activator (uPA or PLAU)	0.252	.043
Matrix metalloproteinase 3 (MMP-3)	0.281	.043
Galectin 3 (Gal-3)	0.221	.044
Monocyte chemotactic protein 1 (MCP-1)	0.199	.045
Neurogenic locus notch homologue protein 3 (Notch 3)	0.170	.045
Carboxypeptidase A1 (CPA1)	0.230	.049

Lower concentration in HFrEF + LBBB	NPX difference	*P*-value
Fibroblast growth factor 2 (FGF2)	0.236	.005
Beta-galactosidase (GLB1)	0.150	.028
Neurabin 2 (PPP1R9B)	0.317	.033
Serine/threonine-protein kinase 2 (SRPK2)	0.205	.036
Serine/threonine-protein kinase 4 (STK4)	0.321	.037
Ableson murine leukaemia viral oncogene (ABL1)	0.211	.045
Mannan-binding lectin serine protease 1 (MASP1)	0.062	.045
Caspase 3 (CASP-3)	0.227	.049
Methylated-DNA–protein-cysteine methyltransferase (MGMT)	0.272	.049

Table 2 gives the full name of all proteins that differed in concentration between HFrEF + LBBB and HFrEF − LBBB. The proteins are separated depending on whether the concentration increased or decreased in HFrEF + LBBB. The *P*-value is adjusted for a FDR of 5%.

HFrEF, heart failure with reduced ejection fraction; LBBB, left bundle branch block.

**Figure 3:**
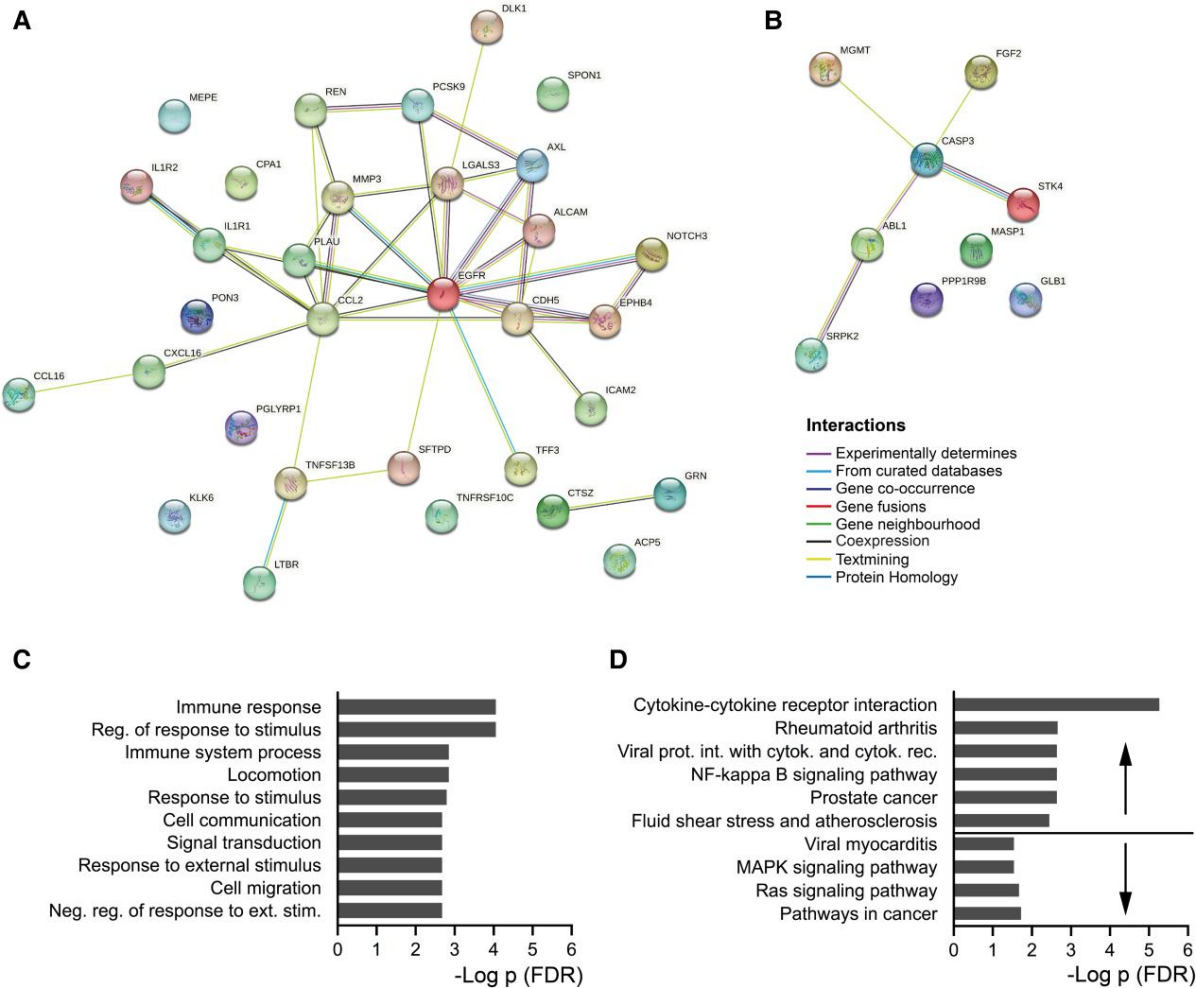
(A and B) Predicted protein–protein interactions of upregulated (A) or downregulated (B) proteins in HFrEF + LBBB compared with HFrEF − LBBB. The basis for interactions, e.g text-mining or experimentally determined, are illustrated with colour-coded connections. The explanation is found at the lower left of the figure. C lists the 10 most significantly affected gene ontology pathways found for upregulated proteins in HFrEF + LBBB compared with HFrEF − LBBB. Downregulated proteins did not affect gene ontology pathways. (D) Affected KEGG pathways, whether upregulated or downregulated proteins were included are indicated by arrows. The *P*-value (*x*-axis C and D) allows for a false discovery rate of 5%. Data were extracted from the STRING database. HFrEF, heart failure with reduced ejection fraction; LBBB, left bundle branch block; GO, gene ontology; KEGG, Kyoto Encyclopedia of Genes and Genomes; FDR, false discovery rate

Protein–protein interactions between the 41 up- or downregulated proteins in HFrEF + LBBB are visualized in *Figure 3A* and *B*. A total of 79 GO pathways were enriched for upregulated proteins and none for downregulated (Supplementary Table S3). These pathways were mainly connected the immune response and cell signalling, e.g. signal transduction and cell communication. The 10 most significantly affected GO pathways are shown in *Figure 3C* and the full list can be found in Supplementary Table S3. Six KEGG pathways were enriched for upregulated proteins, including the nuclear factor kappa-light-chain-enhancer of activated B cells (NFkB) pathway, and four were enriched for downregulated proteins, including the mitogen activated protein kinase (MAPK) pathway where fibroblast growth factor 2 (FGF2), serine/threonine-protein kinase 4, and caspase 3 (CASP-3) were involved (*Figure 3D*). Affected immune-related pathways identified by both GO and KEGG pathway analysis contained changes in both C–C motif ligand 2 and lymphotoxin beta receptor.

## Discussion

In this study of patients with HFrEF, LBBB was associated with a dysregulation of 11% of 364 studied circulating proteins. These proteins were mainly involved in inflammation, immune response and cell signalling.

An increased inflammatory activity correlates with earlier findings from human blood samples^19,20^ and tissues where inflammatory mediators such as osteopontin and tumour necrosis factor α (TNFα) have been found to increase in heart failure with LBBB.^14,15^ Moreover, there are similar results from animal studies.^21^ Whether inflammation has a causative role or is the result of relative ischaemia due to decreased cardiac output needs to be explored further to increase the usability of inflammatory proteins as biomarkers and inflammation as a therapeutic target in heart failure.^22,23^

Fibroblast growth factor 2 and epidermal growth factor receptor (EGFR) were among the most significantly changed proteins in our analysis, where FGF2 was down- and EGFR upregulated in HFrEF + LBBB. FGF2 promotes cardiomyocyte hypertrophy and cardiac fibrosis acting through the MAPK pathway.^24^ Epidermal growth factor receptor is involved in fibrosis through regulating fibroblast differentiation and its deficiency in mice causes heart failure.^25^ In humans, anti-tumour drugs blocking EGFR may increase the likelihood of developing heart failure.^26^ Earlier results from BIOSTAT-CHF imply a role for EGFR both in ischaemic heart failure^27^ and for heart failure patients with diabetes.^28^ Therefore, we would anticipate the up- and downregulation to be inverse, however, since protein content in blood is an indirect way to assess actual changes within the heart muscle this is difficult to interpret. Epidermal growth factor receptor may be cleaved from the cell surface and shed under certain conditions,^29^ which could give an inverse relationship between blood and tissue levels of EGFR. FGF2 is often bound to proteins making up the extracellular matrix.^30^ A change in the FGF2 level in blood could therefore reflect a change in the extracellular matrix composition, rather than an actual change in FGF2 activity. In HFrEF + LBBB, the cardiac remodelling is more pronounced, which is likely to infer large changes to the extracellular matrix composition. The pronounced differences in EGFR and FGF2 concentration depending on the presence of LBBB indicate that these proteins play a role in the pathogenesis of HFrEF + LBBB.

Beyond individual proteins, pathway analysis yielded two interesting findings. Gene ontology pathways regulating the immune response were enriched for upregulated proteins in HFrEF + LBBB, and KEGG pathways connected to MAPK signalling were enriched for downregulated proteins. The downregulation of proteins associated with the MAPK-signalling pathways is more puzzling and does not conform to earlier findings from animal models of HFrEF + LBBB.^8,9,21,31^ The majority of these studies focused on intraventricular differences in protein^9,21^ or RNA^8^ expression in dyssynchrony and resynchronization. That is, a difference between septum and the lateral left ventricular wall, where dyssynchrony leads to a heterogeneity in RNA expression.^8,10^ A difference in intraventricular MAPK-signalling in this setting would perhaps not be reflected in blood samples. As previously mentioned, it is challenging to compare the findings from studies on cardiac tissue protein expression to a study where plasma proteins were analysed. The accuracy of this comparison may increase if the blood is derived from the coronary sinus, which has been performed previously to identify biomarkers in heart failure.^32^ However, coronary sinus blood sampling is invasive, carries an extra risk for patients, and was not available in the current study. However, the repeated findings of affected MAPK-signalling in HFrEF + LBBB indicate that this pathway is important in adaptation to dyssynchronic activation and contraction.

In the current study, we used a protein profiling platform to characterize biological effects of dyssynchrony in HFrEF. Recently, integrated multiomics approaches combining proteomics, metabolomics, and transcriptomics have been used to characterize disease progression and identify biomarkers associated with mortality.^19^ Although beyond the scope of this study, a multiomics approach could in future studies better further the understanding of the biological mechanism of LBBB in HFrEF.

This study has several limitations. The cross-sectional design makes causality uncertain and we can only document an association between LBBB and protein dysregulation in HFrEF. Although propensity score matching was applied and the two study groups matched well, smaller differences in basic characteristics remained. Notably, a medical history of hypertension was less common in HFrEF + LBBB after propensity score matching, and these patients also had a small but significantly lower MAP. Moreover, a lower proportion of patients with HFrEF + LBBB had atrial fibrillation on the ECG taken at the time of blood sampling, but the proportion of patients with a medical history of atrial fibrillation was similar. These differences may affect remodelling and biomarker composition, but we consider it less likely since the difference in MAP was small (5 mmHg) and a history of atrial fibrillation may serve as a more robust marker of this co-morbidity compared with a point-of-care ECG. We did not have access to data concerning which patients received CRT together with repeated blood sampling. Therefore, we cannot determine if the proteome associated with LBBB is reversible with successful resynchronization and processes relevant to resynchronization could not be explored. In this study, we used a proximity extension assay (Olink) to determine protein concentrations of 364 proteins with cardiovascular relevance. This is an accurate method but only proteins included in the assays are evaluated. The panel used focused on proteins with relevance to cardiovascular disease, and differences in proteins not included in the panel will not be recognized. This limits their use as a biomarker discovery technology since only a fraction of proteins are captured and, hence, important proteins for the pathophysiological effect of LBBB may go unrecognized.

Nevertheless, the present study is, to our knowledge, the most comprehensive analysis of protein biomarker expression in patients with HFrEF complicated by LBBB. We demonstrate that LBBB is associated with a significantly altered protein biomarker profile in HFrEF. Pathway analysis showed that inflammation and cell signalling are the main biological pathways involved in HFrEF +LBBB. This could constitute a foundation for discovery of biomarkers with relevance for the disease-modifying effect of LBBB and also function as a foundation for future studies of the biological effects of CRT-induced reverse remodelling by the identification of an amendable circulating proteome induced by LBBB.

## Supplementary Material

xvag009_Supplementary_Data

## Declarations

### Data Availability

The data underlying this article will be shared on reasonable request to the corresponding author.

### Pre-registered Clinical Trial Number

None supplied.
