# Supplementary material for: Protein biomarkers associated with left bundle branch block in patients with heart failure and reduced ejection fraction
Source: ESC Heart Fail. 2026 Jan 13;13(1):xvag009. doi: 10.1093/eschf/xvag009 (PMC13108274; doi:10.1093/eschf/xvag009)
Supplement: xvag009_Supplementary_Data [file xvag009_supplementary_data.zip › Supplement table 1.docx]

**Protein biomarkers – complete list matched comparison**

| **Biomarker** | **p-value (FDR 5%)** |
| --- | --- |
| PSPD | 0.000701 |
| KLK6 | 0.001987 |
| TNFRSF10C | 0.001987 |
| IL1R1 | 0.00476 |
| EGFR | 0.00476 |
| MEPE | 0.00476 |
| FGF2 | 0.00476 |
| CDH5 | 0.006707 |
| ICAM2 | 0.012002 |
| PON3 | 0.012814 |
| CXCL16 | 0.012814 |
| CCL16 | 0.012814 |
| IL1R2 | 0.012814 |
| ACP5 | 0.016358 |
| DLK1 | 0.019223 |
| AXL | 0.019223 |
| EPHB4 | 0.020959 |
| ALCAM | 0.022075 |
| GLB1 | 0.027927 |
| PCSK9 | 0.029985 |
| GRN | 0.030968 |
| TNFSF13B | 0.030968 |
| TFF3 | 0.030968 |
| LTBR | 0.032882 |
| PPP1R9B | 0.032882 |
| SRPK2 | 0.035785 |
| PGLYRP1 | 0.036586 |
| SPON1 | 0.036586 |
| STK4 | 0.036586 |
| REN | 0.036586 |
| CTSZ | 0.036586 |
| PLAU | 0.043264 |
| MMP3 | 0.043264 |
| LGALS3 | 0.043456 |
| ABL1 | 0.045388 |
| CCL2 | 0.045388 |
| MASP1 | 0.045388 |
| NOTCH3 | 0.045388 |
| CPA1 | 0.04854 |
| CASP3 | 0.048576 |
| MGMT | 0.048576 |
| ZBTB16 | 0.051628 |
| METAP2 | 0.056447 |
| MB | 0.056447 |
| LYN | 0.056447 |
| PLAUR | 0.05676 |
| IL1RN | 0.058679 |
| CD93 | 0.061583 |
| IL18BP | 0.061583 |
| CCL22 | 0.061583 |
| MMP2 | 0.062875 |
| DECR1 | 0.062875 |
| SH2D1A | 0.062875 |
| EDAR | 0.066315 |
| CNTN1 | 0.067096 |
| CDKN1A | 0.067096 |
| CD163 | 0.068725 |
| PDGFA | 0.068725 |
| TXLNA | 0.069135 |
| RARRES2 | 0.070841 |
| IRAK4 | 0.070841 |
| DAPP1 | 0.081034 |
| EIF4G1 | 0.081034 |
| HSPG2 | 0.081034 |
| IL6R | 0.085649 |
| TREML2 | 0.085649 |
| SELE | 0.085649 |
| BIRC2 | 0.085684 |
| IRF9 | 0.085932 |
| IL2RA | 0.086743 |
| TNFRSF1B | 0.086743 |
| HCLS1 | 0.086743 |
| S100A4 | 0.086743 |
| TNFRSF11B | 0.094177 |
| PLXNA4 | 0.095117 |
| PRDX5 | 0.095117 |
| HEXIM1 | 0.095117 |
| VIM | 0.097351 |
| ITGB1BP2 | 0.103346 |
| SIT1 | 0.108457 |
| DGKZ | 0.108457 |
| TIMP4 | 0.109288 |
| EPCAM | 0.109288 |
| ITGA6 | 0.109288 |
| DFFA | 0.109288 |
| TANK | 0.109288 |
| GPNMB | 0.110832 |
| PARP1 | 0.110832 |
| IL16 | 0.110832 |
| TNFRSF1A | 0.110832 |
| CCL15 | 0.110832 |
| SCGB3A2 | 0.110832 |
| PRDX1 | 0.110832 |
| CLEC6A | 0.110832 |
| SELPLG | 0.11256 |
| LGALS4 | 0.115358 |
| CXCL1 | 0.116017 |
| NF2 | 0.116923 |
| ANXA1 | 0.116923 |
| IGFBP7 | 0.116923 |
| TRIM5 | 0.117547 |
| MMP9 | 0.118165 |
| SIRPA | 0.118165 |
| LGALS1 | 0.12347 |
| FAS | 0.126369 |
| CPB1 | 0.127063 |
| SERPINA12 | 0.130408 |
| DDX58 | 0.130408 |
| PI3 | 0.132127 |
| BACH1 | 0.138214 |
| PRKCQ | 0.145591 |
| FABP4 | 0.159544 |
| CTSD | 0.159544 |
| DCTN1 | 0.163239 |
| IKBKG | 0.169676 |
| IL18 | 0.169676 |
| PRDX3 | 0.169676 |
| PRSS27 | 0.169676 |
| SH2B3 | 0.172152 |
| CD84 | 0.173997 |
| SPRY2 | 0.174315 |
| IRAK1 | 0.174979 |
| NCR1 | 0.174979 |
| TFPI | 0.183187 |
| MILR1 | 0.186539 |
| ANPEP | 0.18947 |
| SCAMP3 | 0.192203 |
| FADD | 0.197137 |
| CEACAM5 | 0.199475 |
| GDF15 | 0.201839 |
| EGF | 0.202311 |
| SRC | 0.202311 |
| PSIP1 | 0.207079 |
| ICA1 | 0.207648 |
| F2R | 0.210994 |
| CCN4 | 0.211965 |
| MMP12 | 0.225423 |
| TCL1A | 0.225912 |
| IFNLR1 | 0.22654 |
| OLR1 | 0.229185 |
| EGLN1 | 0.23471 |
| CD40LG | 0.267478 |
| IL1RL1 | 0.269384 |
| HNMT | 0.322668 |
| RETN | 0.329571 |
| IL5 | 0.329788 |
| CCL11 | 0.341677 |
| ARNT | 0.364425 |
| HSPB1 | 0.365211 |
| XCL1 | 0.373114 |
| IL17D | 0.373535 |
| SPP1 | 0.381562 |
| CLEC4A | 0.38811 |
| LDLR | 0.390739 |
| HBEGF | 0.393386 |
| VWF | 0.403578 |
| SOD2 | 0.403578 |
| FURIN | 0.410862 |
| IL10 | 0.410862 |
| PPY | 0.411969 |
| IL6 | 0.421448 |
| CCL24 | 0.425091 |
| SPON2 | 0.42638 |
| LGALS9 | 0.42638 |
| THBS2 | 0.42638 |
| TNFRSF14 | 0.42638 |
| NTF4 | 0.433424 |
| GZMH | 0.436529 |
| HAVCR1 | 0.438955 |
| BNP | 0.438955 |
| MPO | 0.438955 |
| S100A11 | 0.438955 |
| TRIM21 | 0.448502 |
| PAPPA | 0.448502 |
| IGFBP2 | 0.448502 |
| F11R | 0.448502 |
| CPE | 0.448502 |
| SMAD5 | 0.454476 |
| AMBP | 0.454939 |
| NFATC3 | 0.46101 |
| SERPINE1 | 0.46101 |
| CCL3 | 0.472717 |
| FCRL6 | 0.474287 |
| PIK3AP1 | 0.480237 |
| IL4R | 0.487167 |
| BOC | 0.487167 |
| TGFBR2 | 0.490102 |
| SORT1 | 0.493937 |
| ADAMTS13 | 0.505634 |
| WIF1 | 0.507499 |
| COL1A1 | 0.513674 |
| MSLN | 0.515713 |
| STC1 | 0.515713 |
| GDF2 | 0.515713 |
| TNFRSF6B | 0.518168 |
| XPNPEP2 | 0.518168 |
| SPARC | 0.523264 |
| VEGFA | 0.525191 |
| AZU1 | 0.530328 |
| LYPD3 | 0.530328 |
| TNFRSF11A | 0.530328 |
| LILRB4 | 0.53114 |
| ITGB5 | 0.533845 |
| THPO | 0.533845 |
| MMP7 | 0.533845 |
| GZMB | 0.534393 |
| F3 | 0.534393 |
| IL1RL2 | 0.539461 |
| IDUA | 0.539461 |
| FXYD5 | 0.539461 |
| GH1 | 0.547171 |
| ITM2A | 0.549374 |
| PRSS8 | 0.549374 |
| NT-proBNP | 0.559886 |
| CSTB | 0.566467 |
| CKAP4 | 0.566467 |
| CXCL17 | 0.566467 |
| DKK1 | 0.566467 |
| CRNN | 0.570841 |
| FCRL3 | 0.575696 |
| ITGB6 | 0.586666 |
| ERBB3 | 0.591573 |
| TEK | 0.592914 |
| CA9 | 0.592914 |
| SELP | 0.593593 |
| CTRC | 0.604205 |
| PRTN3 | 0.608655 |
| CHI3L1 | 0.608655 |
| FAM3B | 0.614341 |
| ANGPT1 | 0.626538 |
| CA5A | 0.632526 |
| DLL1 | 0.63902 |
| KLK11 | 0.640181 |
| CLEC4D | 0.649812 |
| GLO1 | 0.649812 |
| CTSL | 0.651172 |
| FGF21 | 0.651172 |
| ADM | 0.651859 |
| ITGB2 | 0.658992 |
| PRELP | 0.658992 |
| FABP2 | 0.658992 |
| KPNA1 | 0.658992 |
| AGRP | 0.658992 |
| LY9 | 0.663787 |
| CLEC4C | 0.663787 |
| CXCL13 | 0.663787 |
| CXCL12 | 0.663787 |
| GALNT3 | 0.663787 |
| TLR3 | 0.663787 |
| PDGFB | 0.663787 |
| AGER | 0.669827 |
| AREG | 0.669827 |
| PADI2 | 0.671285 |
| IFNGR1 | 0.675392 |
| CNTNAP2 | 0.678057 |
| FST | 0.685804 |
| CD48 | 0.685804 |
| ERBB4 | 0.6863 |
| KRT19 | 0.686558 |
| WFDC2 | 0.690212 |
| CBLIF | 0.690212 |
| LAG3 | 0.690479 |
| NECTIN4 | 0.691228 |
| CDSN | 0.700637 |
| PODXL | 0.700659 |
| LPL | 0.708639 |
| CCL17 | 0.716638 |
| MIA | 0.717175 |
| TNFRSF10A | 0.717175 |
| TNFRSF13B | 0.717175 |
| TNFRSF4 | 0.717175 |
| TNFRSF10B | 0.727099 |
| TRAF2 | 0.727099 |
| KITLG | 0.730322 |
| BLMH | 0.730322 |
| TR | 0.739939 |
| RSPO3 | 0.752542 |
| BTN3A2 | 0.752542 |
| SLAMF7 | 0.752773 |
| TGM2 | 0.770982 |
| IL27 | 0.770982 |
| ICOSLG | 0.772898 |
| KDR | 0.792693 |
| EIF5A | 0.799757 |
| IGF1R | 0.80402 |
| IGFBP1 | 0.80402 |
| HSD11B1 | 0.810553 |
| RET | 0.810553 |
| TGFA | 0.810553 |
| ITGA11 | 0.811693 |
| FLT4 | 0.811693 |
| CLEC4G | 0.811693 |
| CD70 | 0.811693 |
| EPHA2 | 0.811693 |
| IL17RA | 0.811693 |
| CEACAM8 | 0.811693 |
| PGF | 0.811693 |
| KLRD1 | 0.822879 |
| CHIT1 | 0.822879 |
| CLEC7A | 0.825724 |
| PTX3 | 0.825724 |
| TREM1 | 0.825724 |
| CD27 | 0.825724 |
| DPP10 | 0.830676 |
| FCGR2B | 0.831558 |
| HMOX1 | 0.836017 |
| PTH1R | 0.836653 |
| HAO1 | 0.844578 |
| TFPI2 | 0.844578 |
| FGFBP1 | 0.844722 |
| LEP | 0.844722 |
| CD28 | 0.844722 |
| DCN | 0.844722 |
| FOLR1 | 0.844722 |
| FCRLB | 0.844722 |
| IL12RB1 | 0.844722 |
| PECAM1 | 0.847385 |
| DCBLD2 | 0.847385 |
| ESM1 | 0.858852 |
| CEACAM1 | 0.858852 |
| MUC16 | 0.858852 |
| PDCD1LG2 | 0.858852 |
| ACE2 | 0.858852 |
| MDK | 0.868222 |
| ADAM8 | 0.868222 |
| CD160 | 0.868222 |
| FABP6 | 0.868222 |
| CCN1 | 0.868222 |
| KLK13 | 0.877058 |
| VEGFD | 0.880031 |
| LAMP3 | 0.880031 |
| KLK14 | 0.888124 |
| MERTK | 0.890537 |
| ERBB2 | 0.892633 |
| CTSV | 0.897713 |
| GPC1 | 0.899149 |
| TPSAB1 | 0.901033 |
| LY75 | 0.905842 |
| ITGAV | 0.907029 |
| TNFRSF19 | 0.909614 |
| MARCO | 0.909614 |
| PIGR | 0.914456 |
| OSCAR | 0.914456 |
| CD4 | 0.914456 |
| PLAT | 0.915745 |
| CD83 | 0.915745 |
| THBD | 0.915745 |
| MICA_MICB | 0.915745 |
| BMP6 | 0.919569 |
| CD207 | 0.919569 |
| VSIG2 | 0.920311 |
| FOLR3 | 0.920311 |
| ADAMTS15 | 0.920311 |
| HGF | 0.920311 |
| JUN | 0.920311 |
| CXADR | 0.920311 |
| TNFSF13 | 0.920311 |
| FASLG | 0.920311 |
| SEZ6L | 0.921889 |
| KLK8 | 0.921889 |
| FGF23 | 0.937307 |
| NT5E | 0.951717 |
| TNFSF10 | 0.951717 |
| SDC1 | 0.951717 |
| SFTPD | 0.000701 |
| KLK6 | 0.001987 |
| TNFRSF10C | 0.001987 |
| IL1R1 | 0.00476 |

Supplement Table 1 displays a complete list of all 364 individual protein biomarkers analysed in this study. They are ranked after p-value (FDR 5%) obtained from the matched comparison between heart failure with reduced ejection fraction with or without left bundle branch block.
