# Supplementary material for: Protein biomarkers associated with left bundle branch block in patients with heart failure and reduced ejection fraction
Source: ESC Heart Fail. 2026 Jan 13;13(1):xvag009. doi: 10.1093/eschf/xvag009 (PMC13108274; doi:10.1093/eschf/xvag009)
Supplement: xvag009_Supplementary_Data [file xvag009_supplementary_data.zip › Supplement Table 2.docx]

**Protein biomarkers – complete list unmatched comparison**

| **Biomarker** | **p-value (FDR 5%)** |
| --- | --- |
| GLB1 | 0.00214926 |
| TFF3 | 0.00246957 |
| SELPLG | 0.00296062 |
| SIT1 | 0.00327827 |
| KLK6 | 0.00782454 |
| FGF2 | 0.01114611 |
| NT-proBNP | 0.01209679 |
| MB | 0.01209679 |
| PSPD | 0.01209679 |
| PI3 | 0.01209679 |
| CDSN | 0.01209679 |
| F3 | 0.01209679 |
| NFATC3 | 0.01209679 |
| PDGFA | 0.01209679 |
| KLK11 | 0.01448585 |
| PARP1 | 0.01448585 |
| EPHB4 | 0.01590791 |
| MASP1 | 0.01627645 |
| DLK1 | 0.01914392 |
| EGLN1 | 0.02195574 |
| HBEGF | 0.02195574 |
| METAP2 | 0.02195574 |
| DFFA | 0.02195574 |
| MGMT | 0.02195574 |
| SERPINE1 | 0.0247425 |
| TIMP4 | 0.02607444 |
| SRPK2 | 0.02636443 |
| TNFRSF13B | 0.03002982 |
| PRDX1 | 0.03002982 |
| TNFRSF19 | 0.03099557 |
| NF2 | 0.03255355 |
| SOD2 | 0.03942991 |
| IL18 | 0.04341865 |
| OLR1 | 0.04519229 |
| GDF15 | 0.04699334 |
| IGF1R | 0.04902601 |
| TEK | 0.04902601 |
| IRF9 | 0.04902601 |
| IL1RN | 0.04902601 |
| TCL1A | 0.04902601 |
| MEPE | 0.05016383 |
| DCN | 0.05016383 |
| DGKZ | 0.05016383 |
| FURIN | 0.05016383 |
| BIRC2 | 0.05016383 |
| FADD | 0.05016383 |
| GZMH | 0.05033766 |
| CASP3 | 0.05033766 |
| HAO1 | 0.05033766 |
| LTBR | 0.05037031 |
| HSPG2 | 0.05165224 |
| IRAK4 | 0.05165224 |
| BNP | 0.05260087 |
| ADM | 0.05260087 |
| KITLG | 0.05260087 |
| FAM3B | 0.05260087 |
| LPL | 0.05260087 |
| CXCL1 | 0.05260087 |
| TNFRSF1B | 0.05421107 |
| VSIG2 | 0.05911518 |
| AZU1 | 0.06114029 |
| ICA1 | 0.06486164 |
| AGER | 0.06497014 |
| CA5A | 0.06497014 |
| EGF | 0.06571431 |
| TRIM5 | 0.06956261 |
| DECR1 | 0.06956261 |
| REN | 0.06991512 |
| TNFRSF1A | 0.06991512 |
| DDX58 | 0.07088085 |
| PDGFB | 0.07088085 |
| ERBB2 | 0.07894909 |
| RSPO3 | 0.08198037 |
| LGALS4 | 0.08295927 |
| LYN | 0.08475613 |
| IGFBP2 | 0.08601679 |
| RETN | 0.08601679 |
| CCL15 | 0.08601679 |
| IL1R1 | 0.08601679 |
| TREM1 | 0.08601679 |
| PRDX3 | 0.08601679 |
| ZBTB16 | 0.08601679 |
| PSIP1 | 0.09283138 |
| TNFRSF10C | 0.09334398 |
| TXLNA | 0.09334738 |
| NOTCH3 | 0.09464275 |
| CTSZ | 0.09464275 |
| FOLR1 | 0.09464275 |
| HEXIM1 | 0.09695675 |
| CDKN1A | 0.09695675 |
| IL18BP | 0.10018463 |
| TNFRSF11B | 0.10436719 |
| EPHA2 | 0.10674005 |
| CEACAM5 | 0.10674005 |
| EIF4G1 | 0.10841835 |
| FXYD5 | 0.11056633 |
| IKBKG | 0.11056633 |
| IFNLR1 | 0.11197626 |
| CD40LG | 0.11275974 |
| WFDC2 | 0.11336275 |
| FABP4 | 0.11369436 |
| TNFSF13 | 0.12105699 |
| PLAUR | 0.12395782 |
| TGFBR2 | 0.12395782 |
| CTSV | 0.12395782 |
| ITGB1BP2 | 0.12418478 |
| DAPP1 | 0.12418478 |
| BACH1 | 0.13088805 |
| GLO1 | 0.13088805 |
| PIK3AP1 | 0.13088805 |
| SPRY2 | 0.13088805 |
| IGFBP1 | 0.13259714 |
| IRAK1 | 0.13259714 |
| SELP | 0.13320834 |
| PRKCQ | 0.13492289 |
| PPY | 0.13750215 |
| PGLYRP1 | 0.13750215 |
| CD93 | 0.13750215 |
| HCLS1 | 0.13750215 |
| PLXNA4 | 0.13750215 |
| PPP1R9B | 0.13750215 |
| SH2B3 | 0.13750215 |
| ANGPT1 | 0.13939081 |
| TANK | 0.14231589 |
| CXCL16 | 0.14674548 |
| SPARC | 0.14674548 |
| CD27 | 0.14925074 |
| STK4 | 0.1507607 |
| CXCL13 | 0.15343526 |
| KRT19 | 0.15343526 |
| MIA | 0.15343526 |
| ICAM2 | 0.16201962 |
| CDH5 | 0.16281706 |
| TNFRSF14 | 0.16298196 |
| AGRP | 0.16298196 |
| SH2D1A | 0.16298196 |
| CLEC4C | 0.16712296 |
| NECTIN4 | 0.16824337 |
| AMBP | 0.16824337 |
| SEZ6L | 0.16835366 |
| ARNT | 0.16930397 |
| CRNN | 0.17053936 |
| DPP10 | 0.17053936 |
| FGFBP1 | 0.17531675 |
| ABL1 | 0.18022442 |
| SERPINA12 | 0.18022442 |
| THBD | 0.18069537 |
| IL2RA | 0.18979639 |
| TNFRSF10B | 0.20069839 |
| CCL11 | 0.20199297 |
| MMP3 | 0.20510004 |
| XPNPEP2 | 0.20536324 |
| BTN3A2 | 0.20634391 |
| FCGR2B | 0.20639846 |
| IGFBP7 | 0.20673435 |
| GZMB | 0.20673435 |
| LGALS3 | 0.207399 |
| HGF | 0.2100495 |
| PRDX5 | 0.2100495 |
| IFNGR1 | 0.21083891 |
| IL12RB1 | 0.21164433 |
| PGF | 0.21286251 |
| KDR | 0.21286251 |
| CCL16 | 0.21908458 |
| SORT1 | 0.21908458 |
| LYPD3 | 0.21908458 |
| TRIM21 | 0.21908458 |
| ITGA6 | 0.22785903 |
| CLEC7A | 0.22840871 |
| CSTB | 0.23441529 |
| CD160 | 0.23441529 |
| NT5E | 0.24919334 |
| SPON1 | 0.25060517 |
| CEACAM1 | 0.25407565 |
| SCGB3A2 | 0.26954549 |
| CHIT1 | 0.2734546 |
| CHI3L1 | 0.27738195 |
| CXCL17 | 0.27738195 |
| PRELP | 0.27738195 |
| CLEC4A | 0.27946554 |
| TLR3 | 0.27946554 |
| TNFRSF11A | 0.28338011 |
| MMP2 | 0.28893927 |
| CD207 | 0.28942095 |
| SDC1 | 0.28942095 |
| TNFRSF4 | 0.29559577 |
| TGFA | 0.29595276 |
| PON3 | 0.29851588 |
| BOC | 0.29851588 |
| KLK14 | 0.29886457 |
| F11R | 0.29886457 |
| TFPI2 | 0.30179683 |
| PECAM1 | 0.30651053 |
| LILRB4 | 0.31630602 |
| ALCAM | 0.31807744 |
| CD83 | 0.31807744 |
| GPNMB | 0.31807744 |
| FABP6 | 0.31873112 |
| EDAR | 0.31878711 |
| TNFSF10 | 0.32056537 |
| PLAU | 0.3255498 |
| IL6R | 0.33397191 |
| CD84 | 0.33480322 |
| SCAMP3 | 0.33842537 |
| TRAF2 | 0.34874369 |
| AXL | 0.35647617 |
| ANXA1 | 0.36314517 |
| VIM | 0.36798867 |
| SMAD5 | 0.38577984 |
| S100A4 | 0.39055137 |
| CCL17 | 0.3911176 |
| MILR1 | 0.39221095 |
| RET | 0.39221095 |
| DCTN1 | 0.39221095 |
| FGF23 | 0.39876379 |
| KPNA1 | 0.39876379 |
| LAG3 | 0.39876379 |
| STC1 | 0.39876379 |
| LAMP3 | 0.40899388 |
| CKAP4 | 0.41300972 |
| ADAMTS13 | 0.41554291 |
| OSCAR | 0.41785333 |
| IDUA | 0.41785333 |
| FST | 0.42134638 |
| SRC | 0.42877859 |
| TREML2 | 0.42892457 |
| CNTN1 | 0.42892457 |
| RARRES2 | 0.42892457 |
| CD28 | 0.42892457 |
| BLMH | 0.42892457 |
| MMP9 | 0.42892457 |
| IL6 | 0.42892457 |
| THPO | 0.43032017 |
| MMP12 | 0.43291753 |
| DLL1 | 0.43291753 |
| IL1RL1 | 0.44915311 |
| GRN | 0.44915311 |
| ERBB3 | 0.44915311 |
| LGALS9 | 0.45563789 |
| TNFRSF6B | 0.46163541 |
| AREG | 0.46163541 |
| CPE | 0.46163541 |
| CLEC4G | 0.46163541 |
| FLT4 | 0.4664096 |
| TNFRSF10A | 0.48900283 |
| CTSL | 0.49022947 |
| PCSK9 | 0.49814998 |
| CXADR | 0.50684146 |
| NCR1 | 0.50805195 |
| PAPPA | 0.50805195 |
| FCRLB | 0.51443269 |
| ACP5 | 0.52472264 |
| IL17RA | 0.52472264 |
| CBLIF | 0.52472264 |
| EGFR | 0.54425984 |
| GPC1 | 0.54425984 |
| CD70 | 0.54490099 |
| EIF5A | 0.54490099 |
| PTX3 | 0.5515489 |
| F2R | 0.5559982 |
| PDCD1LG2 | 0.5559982 |
| TPSAB1 | 0.56205004 |
| IL27 | 0.56386668 |
| CD4 | 0.56981173 |
| FOLR3 | 0.57886626 |
| PADI2 | 0.57886626 |
| HNMT | 0.57886626 |
| IL17D | 0.58053146 |
| COL1A1 | 0.58169544 |
| MERTK | 0.58169544 |
| LGALS1 | 0.58169544 |
| LEP | 0.59095218 |
| CD163 | 0.59150332 |
| MMP7 | 0.59150332 |
| CCL3 | 0.59657499 |
| MPO | 0.59955109 |
| TNFSF13B | 0.60365533 |
| ICOSLG | 0.60546083 |
| CCL2 | 0.61248226 |
| VEGFD | 0.62244798 |
| GH1 | 0.63450803 |
| FABP2 | 0.63937349 |
| KLK13 | 0.63937349 |
| EPCAM | 0.65254998 |
| PTH1R | 0.65254998 |
| IL16 | 0.65254998 |
| LDLR | 0.65254998 |
| MDK | 0.65254998 |
| SIRPA | 0.66018904 |
| SLAMF7 | 0.66018904 |
| HAVCR1 | 0.66018904 |
| DKK1 | 0.66154462 |
| CCN1 | 0.66173437 |
| XCL1 | 0.67812588 |
| CXCL12 | 0.68137582 |
| KLRD1 | 0.68137582 |
| SPP1 | 0.68319223 |
| ESM1 | 0.68319223 |
| KLK8 | 0.68319223 |
| ADAM8 | 0.68419671 |
| THBS2 | 0.68419671 |
| IL5 | 0.68419671 |
| ITGAV | 0.68755038 |
| LY9 | 0.69182754 |
| FAS | 0.70262262 |
| MARCO | 0.70262262 |
| S100A11 | 0.70262262 |
| MICA_MICB | 0.70262262 |
| CA9 | 0.70658053 |
| VEGFA | 0.71329919 |
| MUC16 | 0.7212017 |
| CD48 | 0.73541 |
| IL4R | 0.73891576 |
| ITM2A | 0.74139571 |
| TR | 0.74139571 |
| PLAT | 0.74919036 |
| IL1RL2 | 0.74919036 |
| ADAMTS15 | 0.75869105 |
| DCBLD2 | 0.75869105 |
| PRSS27 | 0.75869105 |
| vWF | 0.75869105 |
| FGF21 | 0.75869105 |
| CTSD | 0.76028684 |
| GALNT3 | 0.76028684 |
| FCRL6 | 0.76028684 |
| ANPEP | 0.77262 |
| CPA1 | 0.77560592 |
| ITGA11 | 0.77641344 |
| ACE2 | 0.79305223 |
| CCL22 | 0.8087973 |
| CNTNAP2 | 0.81763359 |
| NTF4 | 0.81845295 |
| CPB1 | 0.83025494 |
| TFPI | 0.83025494 |
| TGM2 | 0.83025494 |
| ITGB5 | 0.83743381 |
| CCN4 | 0.8431479 |
| FASLG | 0.8431479 |
| CLEC6A | 0.8431479 |
| CLEC4D | 0.84339825 |
| PIGR | 0.88103202 |
| WIF1 | 0.89572016 |
| PRTN3 | 0.89750568 |
| IL1R2 | 0.89750568 |
| HMOX1 | 0.89750568 |
| ITGB2 | 0.89750568 |
| CTRC | 0.89750568 |
| MSLN | 0.89971484 |
| ERBB4 | 0.90732089 |
| JUN | 0.92096989 |
| LY75 | 0.92096989 |
| IL10 | 0.92096989 |
| SPON2 | 0.92469835 |
| GDF2 | 0.92469835 |
| FCRL3 | 0.92858777 |
| SELE | 0.94726982 |
| ITGB6 | 0.96067014 |
| HSD11B1 | 0.96067014 |
| PRSS8 | 0.96536246 |
| CCL24 | 0.96617999 |
| BMP6 | 0.96617999 |
| HSPB1 | 0.96617999 |
| PODXL | 0.96617999 |
| CEACAM8 | 0.97396117 |

Supplement Table 2 displays a complete list of all 364 individual protein biomarkers analysed in this study. They are ranked after p-value (FDR 5%) obtained from the unmatched comparison between heart failure with reduced ejection fraction with or without left bundle branch block.
