# Supplementary material for: Protein biomarkers associated with left bundle branch block in patients with heart failure and reduced ejection fraction
Source: ESC Heart Fail. 2026 Jan 13;13(1):xvag009. doi: 10.1093/eschf/xvag009 (PMC13108274; doi:10.1093/eschf/xvag009)
Supplement: xvag009_Supplementary_Data [file xvag009_supplementary_data.zip › Supplement Table 3.docx]

**Gene Onthology – affected biological pathways**

| **Biological pathways** | **p-value** |
| --- | --- |
| Immune response | 0.000002 |
| Immune system process | 0.000004 |
| Regulation of response to stimulus | 0.000005 |
| Response to stimulus | 0.000005 |
| Response to stress | 0.000023 |
| Response to external stimulus | 0.000023 |
| Regulation of cell death | 0.000042 |
| Locomotion | 0.000042 |
| Cell migration | 0.000043 |
| Cellular response to stimulus | 0.000059 |
| Signal transduction | 0.000064 |
| Positive regulation of biological process | 0.000064 |
| Cell communication | 0.000070 |
| Positive regulation of neuron death | 0.000093 |
| Regulation of cell population proliferation | 0.000099 |
| Positive regulation of cell migration | 0.000110 |
| Positive regulation of cellular process | 0.000140 |
| Movement of cell or subcellular component | 0.000150 |
| Positive regulation of cell death | 0.000160 |
| Positive regulation of neuron apoptotic process | 0.000160 |
| Regulation of apoptotic process | 0.000180 |
| Regulation of neuron apoptotic process | 0.000190 |
| Cellular response to chemical stimulus | 0.000190 |
| Regulation of neuron death | 0.000190 |
| Response to chemical | 0.000290 |
| Tube morphogenesis | 0.000440 |
| Positive regulation of apoptotic process | 0.000450 |
| Tube development | 0.000510 |
| Protein processing | 0.000520 |
| Positive regulation of response to stimulus | 0.000520 |
| Regulation of cell migration | 0.000550 |
| Regulation of leukocyte activation | 0.000590 |
| Regulation of biological process | 0.000590 |
| Chemotaxis | 0.000700 |
| Cell surface receptor signaling pathway | 0.000700 |
| Regulation of cellular process | 0.000780 |
| Negative regulation of leukocyte activation | 0.000900 |
| Negative regulation of response to stimulus | 0.001200 |
| Regulation of lymphocyte activation | 0.001300 |
| Negative regulation of immune system process | 0.001400 |
| Regulation of signal transduction | 0.001500 |
| Regulation of metabolic process | 0.001500 |
| Regulation of response to stress | 0.001500 |
| Macrophage chemotaxis | 0.001700 |
| Negative regulation of multicellular organismal process | 0.001700 |
| Response to other organism | 0.002000 |
| Negative regulation of biological process | 0.002200 |
| Defense response | 0.002600 |
| Cytokine-mediated signaling pathway | 0.002600 |
| Regulation of phosphate metabolic process | 0.002900 |
| Negative regulation of response to external stimulus | 0.002900 |
| Cellular response to organic substance | 0.002900 |
| Regulation of cellular protein metabolic process | 0.003000 |
| Response to cytokine | 0.003000 |
| Innate immune response | 0.003000 |
| Response to organic substance | 0.003100 |
| Leukocyte chemotaxis | 0.003100 |
| Endothelial cell migration | 0.003200 |
| Leukocyte homeostasis | 0.003600 |
| Regulation of phosphorylation | 0.003900 |
| Interspecies interaction between organisms | 0.004000 |
| Negative regulation of lymphocyte activation | 0.004000 |
| Neurogenesis | 0.004500 |
| Response to tumor necrosis factor | 0.004500 |
| B cell homeostasis | 0.004900 |
| Macromolecule metabolic process | 0.005000 |
| Protein metabolic process | 0.006400 |
| Negative regulation of cell death | 0.006400 |
| Regulation of cellular metabolic process | 0.006500 |
| Regulation of multicellular organismal process | 0.006600 |
| Regulation of response to external stimulus | 0.006700 |
| Cellular response to cytokine stimulus | 0.006700 |
| Regulation of immune system process | 0.007900 |
| Blood vessel endothelial cell migration | 0.008600 |
| Angiogenesis | 0.008900 |
| Generation of neurons | 0.009700 |
| Cell activation | 0.010200 |
| Cell differentiation | 0.010200 |
| System development | 0.010200 |
| Response to reactive oxygen species | 0.010400 |
| Regulation of t cell activation | 0.010600 |
| Regulation of localization | 0.011100 |
| Circulatory system development | 0.011100 |
| Organonitrogen compound metabolic process | 0.011500 |
| Blood vessel development | 0.012000 |
| Negative regulation of cellular process | 0.012000 |
| Regulation of interferon-gamma production | 0.012200 |
| Positive regulation of erk1 and erk2 cascade | 0.012200 |
| Negative regulation of apoptotic process | 0.012300 |
| Regulation of immune response | 0.012500 |
| Regulation of protein binding | 0.012600 |
| Negative regulation of metabolic process | 0.013000 |
| Regulation of inflammatory response | 0.013200 |
| Regulation of cell adhesion | 0.014500 |
| Cell adhesion | 0.014900 |
| Leukocyte activation | 0.015300 |
| Positive regulation of phosphate metabolic process | 0.015700 |
| Cell morphogenesis | 0.015800 |
| Homeostatic process | 0.015800 |
| Monocyte chemotaxis | 0.015900 |
| Negative regulation of notch signaling pathway | 0.015900 |
| Protein phosphorylation | 0.017300 |
| Myeloid leukocyte migration | 0.018200 |
| Positive regulation of protein metabolic process | 0.018800 |
| Negative regulation of immune effector process | 0.019000 |
| Regulation of protein phosphorylation | 0.019700 |
| Regulation of phospholipase c activity | 0.020600 |
| Central nervous system development | 0.021700 |
| Lymphocyte chemotaxis | 0.022600 |
| Regulation of endothelial cell apoptotic process | 0.022600 |
| Cellular response to reactive oxygen species | 0.022800 |
| Regulation of interleukin-1-mediated signaling pathway | 0.022800 |
| Regulation of macromolecule metabolic process | 0.023300 |
| Nitrogen compound metabolic process | 0.023700 |
| Myeloid leukocyte activation | 0.023900 |
| Proteolysis | 0.024800 |
| Nervous system development | 0.024800 |
| Negative regulation of inflammatory response | 0.025200 |
| Phosphorylation | 0.027100 |
| Astrocyte differentiation | 0.027100 |
| Regulation of cell-cell adhesion | 0.027900 |
| Reproductive system development | 0.027900 |
| Cellular process | 0.029300 |
| Humoral immune response | 0.030700 |
| Morphogenesis of an epithelium | 0.031200 |
| Leukocyte activation involved in immune response | 0.032800 |
| Glycosaminoglycan catabolic process | 0.034300 |
| Negative regulation of immune response | 0.034400 |
| Response to bacterium | 0.034600 |
| Anatomical structure morphogenesis | 0.034600 |
| Peptidyl-tyrosine phosphorylation | 0.034600 |
| Regulation of protein modification process | 0.034600 |
| Regulation of cell differentiation | 0.034600 |
| Positive regulation of protein localization to plasma membrane | 0.034600 |
| Response to lipid | 0.036800 |
| Positive regulation of phosphorylation | 0.037000 |
| Cellular response to chemical stress | 0.037100 |
| Angiotensin maturation | 0.037600 |
| Anatomical structure development | 0.037800 |
| Ovulation cycle | 0.039100 |
| Positive regulation of metabolic process | 0.040200 |
| Positive regulation of cellular protein metabolic process | 0.040200 |
| Developmental process | 0.040200 |
| Regulation of t cell proliferation | 0.040200 |
| Animal organ development | 0.040200 |
| Regulation of protein processing | 0.040200 |
| Regulation of natural killer cell differentiation | 0.040600 |
| Anatomical structure formation involved in morphogenesis | 0.040600 |
| Positive regulation of signal transduction | 0.041800 |
| Positive regulation of angiogenesis | 0.041800 |
| Epithelial tube morphogenesis | 0.041800 |
| Regulation of defense response | 0.042200 |
| Cellular response to external stimulus | 0.042200 |
| Neuron projection development | 0.044000 |
| Response to lipopolysaccharide | 0.044000 |
| Neutrophil degranulation | 0.044000 |
| Cellular response to interleukin-1 | 0.044100 |
| Regulation of leukocyte cell-cell adhesion | 0.044600 |
| Neutrophil chemotaxis | 0.046900 |
| Cellular response to growth factor stimulus | 0.046900 |
| Positive regulation of cell population proliferation | 0.047300 |
| Negative regulation of cell-cell adhesion | 0.049900 |

Supplement Table 3 lists all gene onthology biological pathways significantly affected by the 41 proteins found to differ in concentration between heart failure with reduced ejection fraction with or without LBBB. The p-value allows for a false detection rate of 5%.
